# Patellofemoral arthroplasty: expert opinion

**DOI:** 10.1186/s40634-022-00457-z

**Published:** 2022-03-04

**Authors:** Paul Hoogervorst, Elizabeth A. Arendt

**Affiliations:** Department of Orthopedic Surgery, University of Minnesota, 2450 Riverside Ave Suite R200, Minneapolis, MN 55454 USA

**Keywords:** Patella, Osteoarthritis, Indications, Surgical technique, Patellofemoral arthroplasty

## Abstract

Isolated patellofemoral osteoarthritis (PFOA) is a common cause of anterior knee pain in patients over the age of 40 years. Patellofemoral arthroplasty (PFA) is an option to address PFAO when the non-operative or joint preserving management has failed.

The goals of PFA are to reduce pain and increase function of the knee in a bone and ligament preserving fashion while maintaining or optimizing its kinematics. Over the last decades advances have been made in optimizing implants designs, addressing complications and improving functional and patient reported outcomes. Appropriate patient selection has proven to be imperative. Proper surgical technique and knowledge of pearls and pitfalls is essential.

The indications and surgical technique for patellofemoral arthroplasty will be reviewed here.

**Level of evidence:** Therapeutic Level V.

## Introduction

Isolated patellofemoral osteoarthritis (PFOA) is the cause of anterior knee pain in approximately 10–24% of patients over the age of 40 years [9, 26]. It can result in debilitating complaints that severely impact the quality of life. Pain caused by isolated PFOA is typically associated with activities that lead to an increased loading of the patellofemoral joint. Patients report pain behind the kneecap during squatting, lunging, bike riding, stair walking, hill climbing, sitting with the knee flexed for prolonged periods and when rising from a seated position. For isolated PFOA, walking on level ground is rarely affected.

Isolated PFOA is more commonly found in females compared to males due to a higher incidence of underlying pathologies, in particular patellofemoral dysplasia. Iwano et al. reviewed a series of 108 knees in 69 patients with isolated PFOA; 93% were female. McAlindon, et al. [26] also found isolated PFOA to be present more often in females. Within this population, isolated PFOA occurred more than twice as often in females (24%) vs. males (11%). The incidence of combined medial and PF compartment arthritis showed equal incidence between males (7%) and females (6%). In a large multi-center review of 578 patients diagnosed with isolated PFOA, trochlear dysplasia was found to be a strong risk factor, with 78% of patients having a positive crossing sign on the true lateral radiograph. In this study again the majority (72%) of patients were female [10, 16].

Hallmarks of the physical examination include crepitus, pain during active range of motion, a positive Rabot sign, and quadriceps weakness. A lateralized patella is common due to lack of lateral patellofemoral (PF) joint space and can lead to varying degrees of lateral patella tilt and maltracking.

Initial treatment of isolated PFOA consists of activity modification, the use of over-the-counter analgesics, weight reduction, physical therapy, bracing, McConnell taping and injection therapy. Joint preserving surgical strategies include lateral release/lengthening, partial lateral facetectomy, chondroplasty, microfracture, mosaicplasty, autologous chondrocyte implantation and anteromedial tibial tuberosity transfer with the goal of optimizing load-distribution and improving patellar alignment and tracking. Unfortunately, none of these procedures have produced reliable long-term results and are typically considered for patients below 40 years of age. Patellofemoral arthroplasty (PFA) is an option to address PFAO when the non-operative or joint preserving management has failed.

The goals of PFA are to reduce pain and increase function of the knee in a bone and ligament preserving fashion while maintaining or optimizing its kinematics.

Over the last decades advances have been made in optimizing implants designs, addressing complications and improving functional and patient reported outcomes. Appropriate patient selection has proven to be imperative. The indications and surgical technique for patellofemoral arthroplasty will be reviewed here.

## Radiographic evaluation

**Fig. 1:**
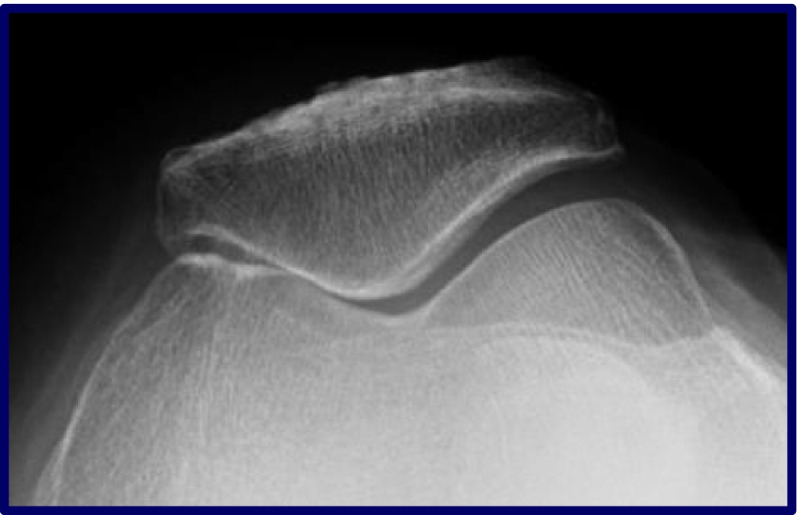
Axial radiograph illustrating medial patellofemoral joint arthritis in a patient 10 years status post a medial tibial tubercle osteotomy and medial imbrication

**Fig. 2:**
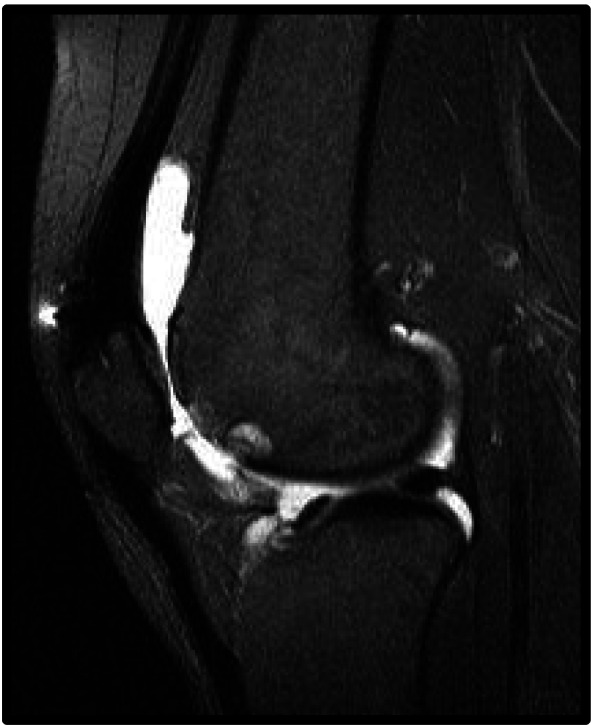
Sagittal slice image of a knee demonstrating central trochlear groove cartilage wear

There are 4 types of PFOA. The most common type is lateral based PFOA which is associated with patellofemoral dysplasia, with or without a history of lateral patellar instability. Medial based PFOA is associated with genu varum, or previous patellar stabilizing surgical procedures such as medializing tibial tuberosity osteotomies or lateral releases (Fig. 1). Global PFOA is associated with primary osteoarthritis (OA) but also post-traumatic OA or systemic entities such as rheumatoid arthritis. Central trochlear groove OA is associated with patients that have high flex demands such as kneeling and jumping activities (Fig. 2).

This pattern can be overlooked on standard sunrise radiographs and are well visualized on magnetic resonance imaging (MRI).

**Fig. 3:**
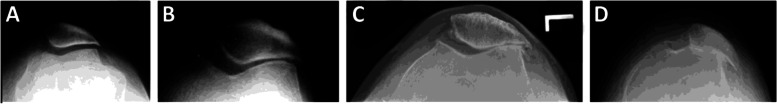
Iwano classification for PF osteoarthritis. **A** Stage I: mild osteoarthritis with joint space at least 3 mm; **B**) Stage II: moderate osteoarthritis with joint space < 3 mm but no bony contact; **C**) Stage III: severe osteoarthritis with bony contact less than one quarter of the joint surface; **D**) Stage IV: very severe osteoarthritis with joint surfaces entirely touching each other, often accompanied by a large lateral patellar osteophyte, and bone loss of the lateral patellar facet

The radiographic severity of PFOA is reported according to the Iwano classification (Fig. 3).

It is important that axial imaging be done in a low knee flexion angle (20° - 30°), which best portrays lateral PF joint space narrowing. It has been shown that patients with evidence of mild patellofemoral arthritis on plain axial radiographs will experience less improvement in pain and function after PFA than those exhibiting more advanced radiographic signs patellofemoral arthritis; cartilage loss based on magnetic imaging alone, without radiographic correlation, have less optimal outcomes [10].

**Fig. 4:**
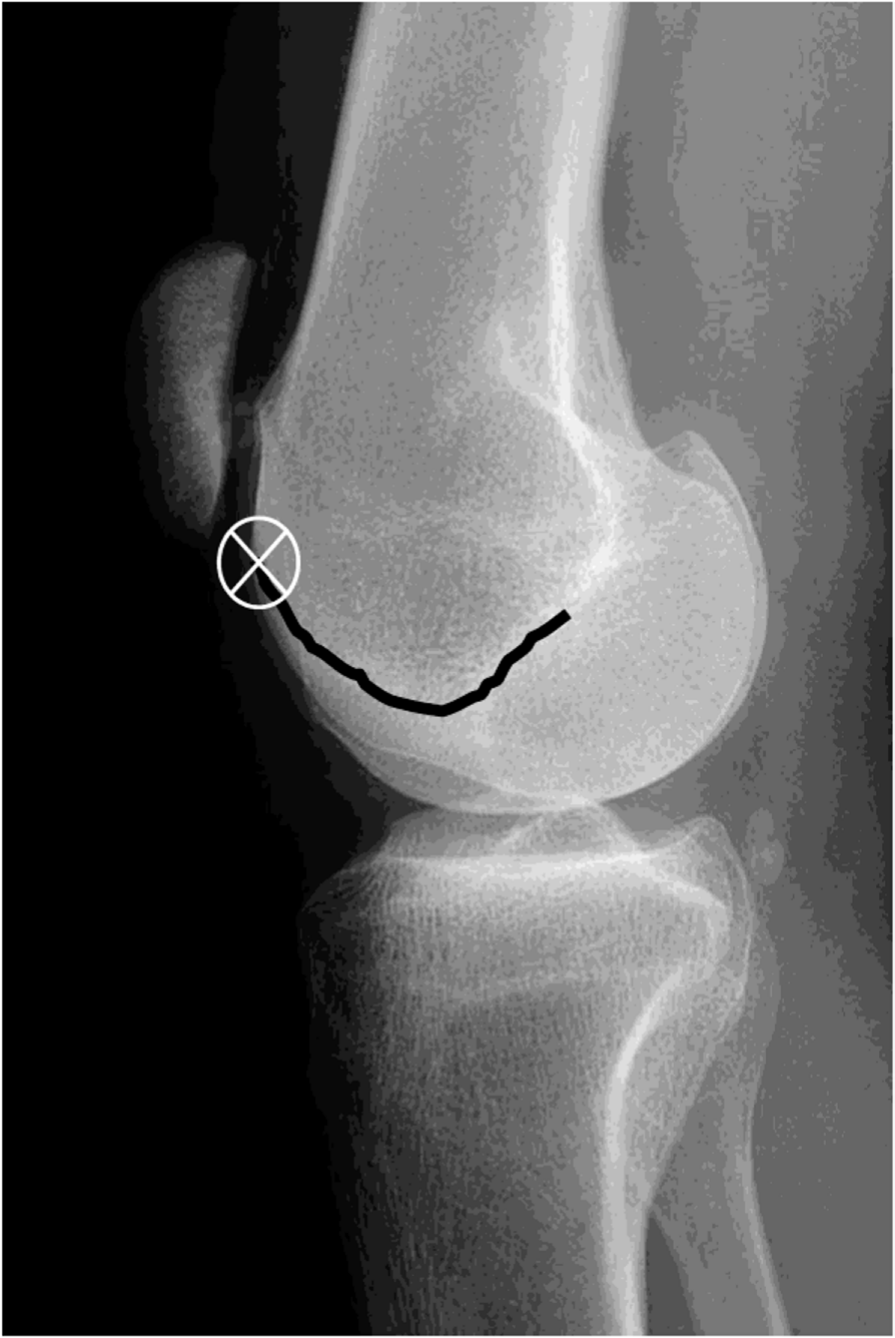
A ‘true’ lateral radiograph of a knee (posterior condyles overlapped). The ‘X’ represents the ‘crossing sign’, where the intercondylar line crosses anterior to the anterior femoral cortex.

The presence of trochlear dysplasia is best identified on a true lateral radiograph (Fig. 4); this is important to identify, as isolated PFOA with trochlear dysplasia has shown to have less progression to tibiofemoral (TF) osteoarthritis [21, 27].

## Indications

The clinical presentation, physical exam findings, and radiographic evaluation should align with the diagnosis of isolated PFOA, and non-operative strategies should be exhausted before a PFA is considered. The most important contraindication for PFA is osteoarthritic changes of the TF joint. MRI and previous arthroscopy imaging can be helpful in evaluating the tibiofemoral joint. Systemic inflammatory disease is associated with involvement of the tibiofemoral joint and therefore considered a contraindication as well [33].

Iwano stages I/II as well as a flexion contracture of > 10 degrees is associated with poorer outcomes [22]. Patella baja with a Caton-Deschamps Index (CDI) of < 0.8 is contraindicated [12]. Relative contraindications include a high knee flexion, depending on patellar height. Both can be associated with an increased chance of articular damage and synovitis due to the patellar component engaging the residual native trochlea [22, 23].

Distal femoral osteopenia should be considered a relative contraindication [36], since most PFA designs utilize peg fixation into distal femoral cancellous bone.

Lower extremity alignment typically is an indication of varying degrees of tibiofemoral cartilage wear. Limb alignment of > 5 degrees of valgus and > 3 degrees of varus has been cited a contra-indication [22, 23].

PFA can be considered in patients ideally between 40 and 65 years old. Although the PFA may be a patient’s final implant it needs to be discussed that progression of OA of the tibiofemoral joint may result in conversion to a total knee arthroplasty (TKA).

## Designs

The first designs of PFA were inlay or resurfacing designs aiming to replace the worn cartilage of the trochlea and patella. These implants did not address anatomical abnormalities such as patellofemoral dysplasia which is common this patient population. Therefore, these implants were associated with high rates of revisions due to patellar maltracking, luxation and persistent pain. Inlay prosthesis have been successful for central grove OA in the absence of anatomical abnormalities. Patients with high-normal patellar height index or patella alta, as well as a craniolateral type of arthritis with additional lateralization, should be considered contra-indicated for an inlay technique PFA [3].

Second and third generation PFA designs are referred to as onlay or trochlear cutting designs. The more recent designs have radius of curvature similar to those of TKAs, a larger anterior flange and thinner lateral edge to optimize patellar kinematics. These onlay designs are helpful in addressing isolated PFOA due to underlying trochlear dysplasia since the anterior femoral cut eliminates this. The onlay designs allow for a more optimal proximal realignment reducing the tibial tuberosity – trochlear groove (TT-TG) distance therefore avoiding the necessity for additional osteotomies of the tibial tuberosity [35].

The second and third generation PFA are reported to have satisfactory patient reported and functional outcomes and reduction in mechanical complications [2, 4, 11, 28].

## Surgical technique

Besides adequate patient selection, optimal implant positioning and surgical technique are important factors to optimize the functional outcomes, pain relief and long-term survival of the PFA.

PFAs are routinely done in an outpatient setting in our center (University of Minnesota, MN, USA).

The PFA procedure begins with the patient in supine position with a bump under the ipsilateral buttock to internally rotate the operative extremity so the patella faces up. A non-sterile tourniquet is placed but not inflated until cementation of the final implant. A positioning aid such as the De Mayo or Alvarado leg positioner is typically used. Foley catheters and drains are not routinely utilized.

General or short-acting spinal anesthesia techniques are employed, but the latter is preferred as this allow for early mobilization. For post-operative pain control a circumferential peri-articular block is placed intra-operatively. Though researched in TKA patients, the added benefit of an adductor canal blocks in combination with the peri-articular block remains unclear [1, 37]. We do not recommend the use of a femoral nerve block as this may result in quadriceps weakness and subsequent delay in mobilization and increased risk for falls.

### Approach

**Fig. 5:**
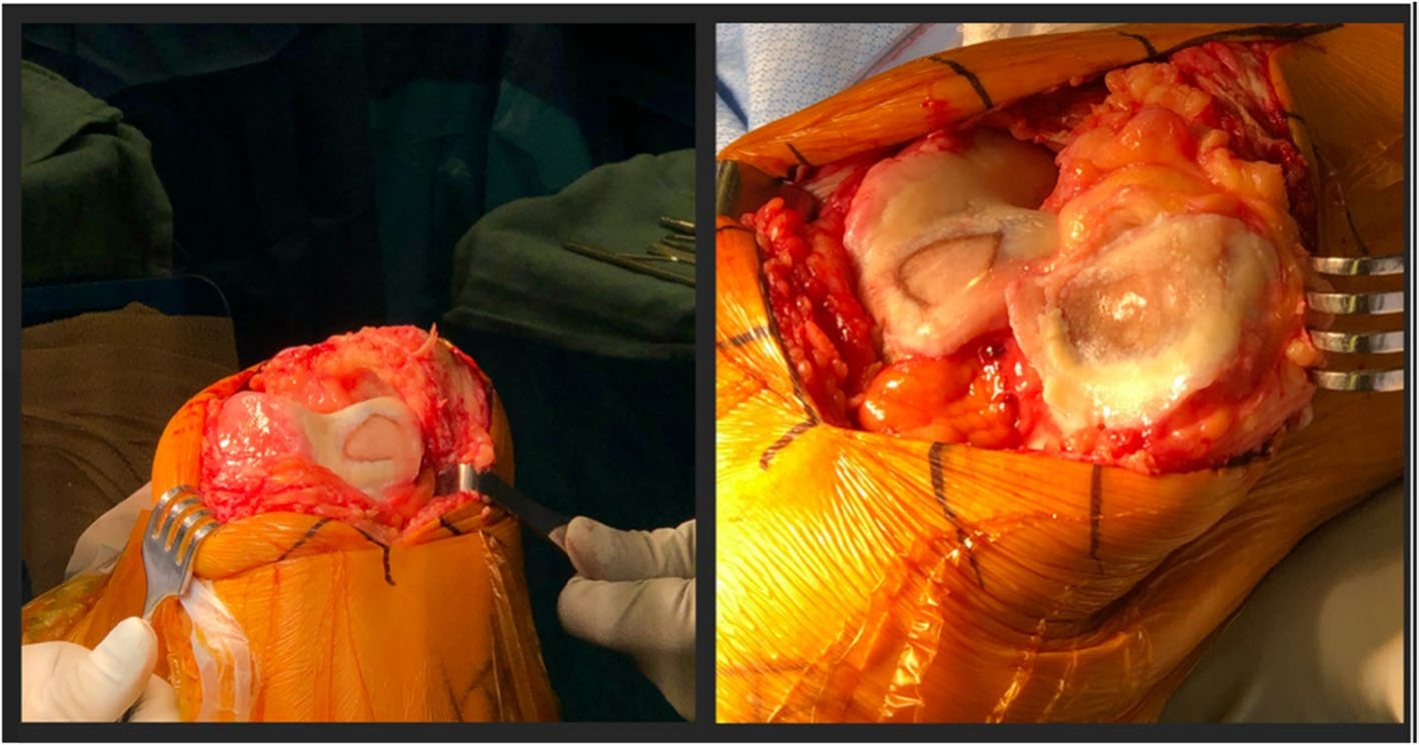
Intra-operative image depicting a lateral based isolated patellofemoral osteoarthritis addressed via medial parapatellar approach

The operative approach consists of a standard midline incision, similar to the approach for a TKA (Fig. 5). Previous surgical scars are incorporated when possible. Standard medial parapatellar approaches are most commonly used, although mid-vastus [32], sub-vastus and lateral arthrotomies [30] are described. The lateral approach can be considered in pre-operative weakness of the vastus medialis obliques (VMO) or when the patella is significantly translated laterally and the need for an extensive lateral release is anticipated. This approach preserves all medial vascularization to the patella. Reports on superiority of the medial versus lateral approaches are ambiguous as functional outcomes are similar, but the lateral approach seems to improve patellar tilt [18, 30].

.Preservation of the meniscus and intra-meniscal ligament is important during the approach followed by careful inspection of the medial and lateral tibiofemoral compartments regarding cartilage wear. If present, conversion to a TKA should be considered.

### Femoral component positioning

Different manufacturers offer PFA systems, and each has unique instrumentation to aid in correct bone resection and implant positioning. It is imperative to be aware of the specific characteristics of the instrumentation and implants in order to successfully replace the patellofemoral joint.

Onlay PFA systems rely on an appropriate anterior femoral cut that is flush with the anterior cortex. Accurate positioning is aided by the anterior trochlear femoral resection guide. The goal is to achieve a cut that allows for placement of the femoral component of the PFA in an anatomic or kinematic alignment. Two anatomic landmarks that can assist in proper orientation of this cut are Whiteside’s line (the deepest part of the trochlear sulcus) and the trans epicondylar axis (TEA) which indicates the native distal femoral external torsion/rotation. The anterior cut should be made perpendicular to Whiteside’s line. This may be difficult to reliably identify in severely dysplastic patellofemoral joints. The anterior cut should be made parallel to the TEA.

**Fig. 6:**
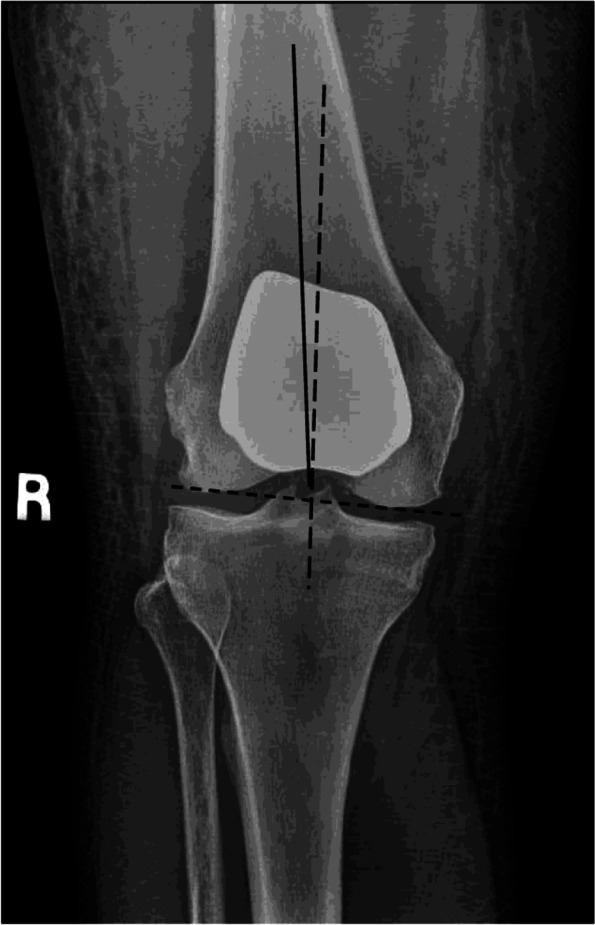
PFA femoral component positioned in line with the distal femoral joint line (dotted line) and not in line with the femoral axis (solid line). This places the femoral component in a varus position

Next, appropriate orientation of the femoral component in the coronal plane is essential to ensure adequate engagement of the patellar component into the femoral component during the early phases of flexion. System specific instrumentation is available to achieve this. The goal is to align the component with the anatomical axis of the femur. This will limit the chances placing the component in varus which can result in a patellar clunk and patellar hop in early flexion as proximal portion of the femoral component is too medialized, and the patellar component does not enter the trochlear groove appropriately. Care must be taken when there is femoral valgum due to an hypoplastic lateral femoral condyle. Using the axis of the femoral shaft, and not the distal femoral joint line, will minimize this potential complication (Fig. 6).

Lastly, adequate medial to lateral coverage of the femoral component is important and any medial or lateral overhang of the component should be avoided. If necessary to achieve this, undersizing of the femoral component is advised. The lateral edge of the femoral component should closely match the lateral part of the anterior cut. This reduces the TT-TG distance. The transition from residual native femoral cartilage to the femoral component should smooth to prevent impingement. If one cannot match the femoral component to the native cartilage, recessing the medial side of the component is preferred over any protrusion of the lateral side of the component, which will result in patellar catching in deep flexion to extension.

There are cases where this is not fully achieved. In those situations, one can change the axial rotation of the anterior cut which allows for the coronal groove to be altered to lie more closely with the native groove line without compromising the prosthesis cartilage transition [7]. Flexion of the femoral component is also a strategy proposed to obtain a flush surface distally. This should be carefully considered as this can lead to catching when the patellar component engages the femoral component in patients with patella alta or when a prosthesis with a short femoral flange is used.

### Patellar resection and positioning

The goals of placement of the patellar component are to restore native patellar thickness without overstuffing, avoid patellar tilting and ensure appropriate patellar tracking.

With a caliper the patellar thickness is measured prior to resection. The patellar component should be placed parallel to the trochlear groove of the femoral component using resection guides or in a freehand manner. Orientation of the proper resection plane may be difficult as a result of lateral patellar facet bone loss or patellar dysplasia resulting in a vertical medial facet (Wiberg type IV patella). Significant bone loss can occur especially lateral on the patella. To manage this a lateral facetectomy can be performed. Residual patellar thickness ideally remains > 12 mm to minimize the risk of patellar fractures. Both symmetric and asymmetric dome-shaped components can be used. To aid in optimizing the tracking of the patella it can be medialized, and in the setting of a patella alta it can be placed more distally. Alternatively, in the setting of patella alta, a PFA design with a longer anterior flange can be selected. It is noted that patients with patella alta are at a higher risk for failure of the PFA [14].

### Trial component evaluation

Implant position should be carefully evaluated with the trial components in situ. Temporary and partial closure of the arthrotomy with sutures or towel clamps can aid in evaluation of patellar tracking and implant position. It is important to assess the engagement of the patellar component and the femoral component during extension to early flexion, and deep flexion to extension. The patella should track smoothly throughout range of motion.

If a problem with patellar tracking is observed, the orientation and sizing of the implants should be reassessed and changed or repositioned as necessary. When utilizing a medial parapatellar approach, a lateral release can be considered to aid in patellar tilt or tracking issues. Lateral release should not be relied upon to correct lateral patellar subluxation if noted when trialing implants. In this case, component repositioning is preferred. If this does still not allow for appropriate tracking a tibial tuberosity osteotomy should be considered.

### Cementing and closure

Once satisfied with both the sizing and orientation of components, as well as the patellar tracking and range of motion the tourniquet is inflated. Care is taken to flex the knee > 90 degrees so the quadriceps is flexed in order to reduce the chance of the tourniquet disturbing the patellar tracking. Next, the trial components are removed and the knee is irrigated with pulsatile lavage. The cancellous bone surfaces are then dried. Starting with the femoral component the definitive implants are placed and all excess cement is removed. After cementation the tourniquet is released. The subcutaneous and skin layer is closed according to the surgeon’s preference.

### Rehabilitation

Postoperative antero-posterior (AP), sunrise and lateral radiographs are obtained to evaluate implant sizing, orientation and fixation. The femoral component should be oriented in valgus on the AP view and flush with the anterior distal femoral cortex on the lateral. On the axial view the patella is centered in the trochlea with neutral tilt.

Immediate weightbearing is allowed as tolerated with the assistance of crutches or a walker. Multimodal pain control is initiated. Physical therapy will assess safe ambulation and will focus on gait, strength and range of motion exercises.

Although the level of evidence is low, return to sports is possible. For some patients, activities that involve high flexion is uncomfortable, even if motion is restored (rock climbing, some yoga positions). It is however not recommended to partake in high impact and jumping sports such as basketball, soccer, volleyball, and mixed martial arts [13]. For most patients, the return r to their previous sports levels with greater comfort and resilience is an attainable goal.

## PFA vs TKA

An alternative to PFA is the placement of a TKA It is generally accepted that with increase age patients with isolated PFOA would benefit from a TKA as the likelihood of concomitant tibiofemoral OA rises. However, there is ample debate whether younger patients should undergo PFA or TKA in this setting. Common arguments in favor of PFA are that it is believed to be a quicker procedure and recovery, more bone sparing with more optimal postoperative knee kinematics and that it is technically not challenging to revise to a TKA when needed. Indeed, using the PFA as a (potential) staging operation in the 40–65 years old age group is a reasonable option in otherwise healthy individuals.

Arguments in favor of the TKA include the higher rate of revisions after PFA and thus lower survival rates found in national registries. The 5-year cumulative percent revision of PFA for any reason ranges from 8.0% (95% CI 4.5 to 11.5) in Norway to 18.1% (95% CI 15.5 to 20.7) in the Netherlands [24].

Other arguments for TKA include the reported higher rate of complications [5].

A recent systematic review and meta-analysis comparing PFA and TKA by Peng et al., included 3 randomized controlled trials (RCT) [15, 19, 28], of which only two reported clinical outcomes [19, 28]. They found that throughout the first 2 years postoperatively, a statistically significant higher activity level, and better functional recovery were observed for PFA compared with TKA [29]. They do not report whether this statistical difference also translates into to a clinically relevant difference. The authors did not find statistically significant differences in complications, revision rates, and satisfaction rates between the two procedures [29].

.When analyzing the individual RCTs, Joseph et al. included 60 patients with an average age of 64 years. They did not observe a statistically or clinically significant difference in any domain of the WOMAC, UCLA, Oxford Knee Score (OKS), American knee society score (AKSS), EQ-5D and EQ-VAS at 12 months. No differences in complications or revisions were observed. Mid-term follow up at 2 and 5 years by means of OKS and EQ-5D did not reveal any differences either [19].

Odegaard et al. evaluated 100 patients with a mean age of 64 years for SF-36, OKS, knee injury and osteoarthritis outcome score (KOOS) and complications. They that found patients undergoing PFA obtain a better overall knee- specific quality of life than patients undergoing TKA throughout the first 2 years after operation. At 2 years, only KOOS function differed in favor of patients undergoing PFA whereas other dimensions do not show a difference between groups. No differences in complications or revisions were reported [28].

Lower-level evidence found statistically significant higher mean forgotten joint scores (FJS) in patients undergoing PFA compared to those with TKA at 1 year [25]. This mean difference was 7.3 points, which may not be clinically relevant as it does not meet the threshold for a minimally clinically important difference (MCID) [8, 17].

Kamikovski et al. retrospectively evaluated 23 PFAs and 23 TKA and found both statistically significant and likely clinically relevant differences in the KOOS at 1 year and 2 years follow up, but no clinically significant differences in WOMAC at 2 years. UCLA scores were similar at both time points [20]. The small number of patients make it difficult to extrapolate these results.

Reports on cost efficiency of PFA compared to TKA are equivocal and do not consistently favor one over the other [6, 15].

### Future directions

Although much knowledge has been gained over the past decades regarding implant design, PF joint biomechanics and patient selection, further research is needed. Larger prospective cohort studies and RCTs could help in further discerning which patient with isolated PFOA would benefit most from a PFA. Besides functional outcome scores more uniform data on patient satisfaction should be considered as this was found to be limited and highly variable [33].

Innovations such as the use of robotics and navigation in the placement of PFA needs to be analyzed as early retrospective data appear to be encouraging [31, 34].

## Expert opinion

Patellofemoral arthroplasty (PFA) is a viable option to reliably address isolated PFAO when the non-operative or joint preserving surgical management has failed.

The goals of PFA are to reduce pain and increase function of the knee in a bone and ligament preserving fashion while maintaining or optimizing its kinematics. Over the last decades advances have been made in optimizing implants designs, addressing complications and improving functional and patient reported outcomes. Appropriate patient selection has proven to be imperative. Patients < 65 years with isolated PFOA due to underlying anatomical abnormalities such as trochlear dysplasia, patellar maltracking and post-traumatic osteoarthritis are good candidates. The use of third generation onlay PFA designs are recommended. Proper surgical technique and knowledge of pearls and pitfalls is essential.

## Data Availability

NA
